# 

**Published:** 1894-09

**Authors:** 


### TABLE OF CONTENTS.

ORIGINAL COMMUNICATIONS. PAGE

Duplex Obturator. By DR. J. W. RHONE, Bellefonte, Pa. . . . . . . . . 545

The One Material. By MAX GREENBAUM, D.D.S., Philadelphia . . . . . 550

A New Era in Dental Practice. By W. G. A. BONWILL, D.D.S., Philadelphia . . . . . . . . . . . . . . . . . . . . . . . . . . . 562

REPORTS OF SOCIETY PROCEEDINGS.

New York Odontological Society . . . . . . . . . . . . . . . . . . . 575

Odontological Society of Pennsylvania . . . . . . . . . . . . . . . . 580

EDITORIAL.

The Recent National Conventions . . . . . . . . . . . . . . . . . 595

The Present Status of Dental Knowledge in the Medical Profession . . . . 598

No Sex in Science . . . . . . . . . . . . . . . . . . . . . . . . . 602

BIBLIOGRAPHY . . . . . . . . . . . . . . . . . . . . . . 602

OBITUARY.

Richard Bayly Winder, M.D., D.D.S. . . . . . . . . . . . . . . . . 604

Resolutions of Respect to Dr. Winder . . . . . . . . . . . . . . . 605

DOMESTIC CORRESPONDENCE.

Oristry: Another Name . . . . . . . . . . . . . . . . . . . . . 606

NOTES AND COMMENTS . . . . . . . . . . . . . . . . . . . 607

CURRENT NEWS.

Missouri State Dental Association . . . . . . . . . . . . . . . . . 608

First District Dental Society of Illinois . . . . . . . . . . . . . . . . 608

